# The Embryo Physician as a Specialist

**Published:** 1888-08

**Authors:** T. H. Nott

**Affiliations:** Goliad, Goliad County, Texas


### THE EMBRYO PHYSICIAN AS A SPECIALIST.*

BY T. H. NOTT, M. D., GOLIAD, GOLIAD COUNTY, TEXAS.

*Mr. President and Fellows of the Texas State Medical Association:*

This doubtless seems a singular caption for a report from your chairman on Practice of Medicine, Pathology and Therapeutics, but before judging me too severely allow a word of explanation.

In the first place I am an isolated member of this honorable body, there being no other member in less than seventy-five or one hundred miles of me, and no medical organization nearer than that, while I am without railroad or telegraphic communication with the rest of the State or world.

To get up a report of the advances made in the important branches assigned to me, under such circumstances, would be simply to cull the journals which you have read, or to give an egotistical report of my own limited experience, or to worry my brain and consume your time over fine-spun theories not yet proved by anybody.

Again, I claim that the subject chosen is at this day and time the very fittest to engage the attention and study of your Section on Practice, Pathology and Therapeutics. If I understand my duty aright, it is more to suggest those things which will the most surely and rapidly advance these branches, and which are not generally understood by the busy country practitioner, than to give a resumé of those facts and principles which have brought medicine to its present advanced position.

*Read before the Texas State Medical Society.

If this is the correct idea of our duty, then I could surely select no subject which should more seriously engage our attention than the one presented. The first point in order will be to define my subject. This may best be done by comparing what *was* and what *is* a specialist.

In the good old times gone by, a specialist was "a physician and something more." A physician who, having grown ripe in years, in knowledge and in experience; who, having learned all that his co-laborers and general practical experience could teach him, and not yet being satisfied, singles out that branch for which he has proved himself best fitted and in which he has been most successful, and by concentrating his energies, backed by a ripe experience, he pushes forward into paths before untrodden and opens up new fields of labor as far in advance of the general practitioner as the electric light is of common gas.

This gentleman is what *was* a specialist; and now what *is* a specialist? As our late retiring President of the American Medical Association happily expresses it, "Something less than a physician." A sort of one-horse doctor. Not a man who, having proved himself a physician, has a right to select that branch for which he finds himself best suited, but one who guesses that he will make a good oculist, gynæcologist or dermatologist, as the case may be. In not a few instances his father guesses for him that he was born an oculist. Accordingly an oculist is selected as his preceptor. He finally graduates at college by scratching through on six branches and taking the highest prize on the branch selected for his future specialty. He is now put into an eye-hospital at home for a year, then sent abroad to attend the eye-clinic of Donders, Wells, Stelwag, or some other foreign celebrity, and then returns to some of our cities to practice ophthalmology. He knows a Bright's retina, choked disk, gray atrophy, blue atrophy, etc.; can talk and write learnedly of far points, near points, angles and technicalities that few of us ever heard of, and he can use a knife beautifully, for he has spent hours practicing on pig's eyes and sheep's eyes, and polished off with rabbit's eyes, which, I believe, are said to be most difficult to operate upon. He brings letters of recommendation from several foreign professors and from his old tutor at home. He settles in a town of twenty or thirty thousand inhabitants, which is crowded with several dozen physicians, but which will only support one oculist, and he has the cheek to ask and expect all the physicians of his city and all the surrounding towns to send him their eye cases and to drum for him. He sends his advertisements to the country papers around and has them printed in two or mȏre languages. He sits back on his dignity and grows rich and famous while we struggle for a living and a small reputation and drum for him. But why is it that he thus deceives us and the people? For two reasons—first, most of us know so little about the eye, and second, because he operates so beautifully we take it for granted that he knows the rest. Because he can extract a cataract, straighten a crossed eye or do an irridectomy, we believe him to be an experienced oculist. But, gentlemen, be not deceived; he is a born specialist. He knows nothing but the eye, and therefore cannot know the eye. He has studied it as he would a mistletoe bough without knowing anything of the tree or the atmosphere whence it derives its sustenence. True, he operates beautifully; he learned it at Wien, or Paris, or Berlin, on pigs' eyes. But does he know when to operate, or perhaps better, when *not* to operate? And outside of the knife what does he know? What of malaria, neuralgia, indigestion, torpid liver, brain diseases, uterine diseases and a hundred other diseases and conditions which affect the eyes and require treatment before the eye trouble can be relieved?

To the general practitioner, ocular therapeutics may suggest a blue mass pill, twenty grains of quinine, hydrocloric acid, potassium bi-carbonate, iodides, etc., as the eye trouble proceeds from derangement of the liver, malaria, indigestion, brain diseases, etc. To the oculist born, ocular therapeutics at once suggest atropine, eserine, boracic acid, together with knives, hooks and spoons of various shapes and sizes. Uterine therapeutics to our older gynæcologists may mean the major portion of our general therapeutics, while to the gynæcologist born it alludes only to nitrate of silver, acid chromic, iodoform, carbolic acid, specula, tenacula, applications, curettes, needles, silver-wire, etc.

To our older laryngologists, the therapeutics of the larynx and pharynx may suggest cod liver oil, wine, mountains, Colorado or Florida. To the laryngologist born, it at once suggests mops, swabs, sprays, insufflators, respirators, etc. A good example of this sort was furnished me on my last visit to one of our Northern cities. The good old specialist, a man of deservedly fine reputation, was for some cause absent from the clinic, and his assistant, a born laryngologist of twenty-three or twenty-four years, took charge of the clinic in the most dignified manner. The first two cases presented were two men who had not attended previously, and therefore required careful examination to make correct diagnosis. They were asked no questions, but immediately seated in front of a bright gas-jet, the specialist in front with his brow-glass well polished and adjusted, tongue depressor and laryngeal mirror cleansed and warmed, patient's head inclined at a certain angle, mouth opened, light reflected, tongue depressed and laryngeal mirror in position, image caught and diagnosis at once rendered, together with prognosis.

Both were pronounced granular or glandular laryngitis of long standing, and would require a long time to cure, but the patients were told that if they would attend regularly three times a week for six months they would both be well at the end of that time.

Not feeling satisfied with what I had seen and heard, I followed these two young men out of the room, when I had an opportunity of making a more thorough, satisfactory examination ; their histories were short and clear. They were brothers, one between twenty and thirty years of age and the other between thirty and forty ; the elder had been in bad health for two years, the younger for one year. Both coughed a great deal, and expectorated large quantities of pus, particularly on rising in the morning ; their parents both died of lung trouble. I applied my ear to the chest of the older man and heard loud cavernous breathing; percussion also revealed a large cavity. I kept my own counsel, but was still more thoroughly convinced of the impropriety of attempting to make men specialists from birth, I may say. This also gives a very clear idea of therapeutics and pathology as seen through the eyes of the young specialist.

Now, gentlemen, I would ask, can practice, pathology or therapeutics be advanced by such men? What are their fine-spun theories worth? Is it not lowering the standard of medical education? Are such men entitled to an M. D. after their names? If one man takes the eye, another the ear, another the brain and spinal cord, another throat and larynx, another genito-urinary diseases of the male, another genito-urinary diseases of the female, another diseases of the chest, another diseases of the bones and deformities, another the teeth, another diseases of children, then there is nothing left for the general practitioner but the abdominal viscera (nothing but guts) to study. Is it possible to divide up so complex a machinery as the human system and understand one part *ab initio*? When we have studied it as a whole for years, and then isolated a single organ or system as our special study, it is with difficulty that we partially comprehend its workings, and impossible to explain many of its diseases and their rational treatment; what, then, can a man know of any part of the human system who has not studied the whole?

Again, I would ask, if the first generation of young specialists, who have been tutored by our old specialists, who were general practitioners, furnishes such examples as these, what, for heaven's sake, will be the type of the second and third generations of specialists, who must be tutored by these born specialists? Fortunately for mankind, this in-and-in breeding would soon extinguish the species; but, alas, for our profession, we must suffer so severely from the reaction and be thrown back a quarter or half century and begin again to train general practitioners before we can have the material out of which to make a specialist worthy the title. This, gentlemen, must be our fate, unless we at once discontinue this ruinous practice of attempting to born specialists, while we yet have an abundance of material among our experienced general practitioners to make specialists who will be blessings to mankind, honors to our profession and leaders and teachers in every branch of medicine. Why, gentlemen, I see from a recent number of the New York *Medical Record*, where, at a collation of the dentists in Cincinnati or Chicago, one of their profession predicts that the time is near at hand when a man will not be allowed to practice dentistry without a thorough medical education. Only yesterday a gentleman of high culture (an Episcopal Bishop) said to me: "I am afraid of a young specialist." Now, if the dentists and laymen are becoming afraid of born specialists, how much more should we be afraid of them, who know so well what they are? Can we remain so blinded to the interests of our noble calling as not to see defects so patent to the outside world? Can we afford to see our standard thus lowered while we are pretending to be so clamorous for a higher standard? Dr. G—— very happily fits Bacon's definition of a clever man to that which a specialist should be—a man who knows something about everything and everything about something. The sooner we accept this as our standard the sooner we realize the fact that a specialist should be something more than a physician—the sooner will we be prepared to raise our standard of medical education. But so long as we allow ourselves to be deceived and accept as our superiors men who are much less physicians, just so long will we retard our progress and retrograde our standard. In conclusion, I would say, if there are any young specialists present, that, while I intend nothing in the least degree personal, still I make no exceptions in any one's favor. The principle is radically wrong, and I am opposed to it, and hope that our young specialists will see the error of their ways before it is too late, and turn to the general practice of medicine, and make physicians of themselves before they try to become specialists.

And, now, while our illustrious President and this honorable body are, from a spirit of philanthropy, using their utmost endeavors to procure legislation on hygiene, quarantine, regulating practice of medicine, board of health, home for inebriates, etc., etc., let us not forget the mass of our people who are afflicted in the line of some specialist. Let us put in a plea to keep our people out of the hands of the born specialist, by inserting a clause in the article regulating the practice of medicine, which shall forbid any person to practice a specialty in this State who has not done a general practice for at least ten years.

As to the admitted ignorance of the general practitioner concerning special diseases, it is very much like the assumed knowledge of the young specialist, viz., more imaginary than real, and while it would require years for the young specialist to acquire that general experience which is so indispensable to a man who proposes to make a specialist of himself, it would require but a few months for the man who has already toiled for his experience, and who knows for what branch he is best fitted, to brush off his supposed ignorance and acquire all that is known on any special branch. A few months' private course in a special hospital under an old specialist will make a better dentist, gynæcologist or laryngologist out of an experienced general practitioner than the man who enters the profession as a specialist can make in a lifetime.

Again, we are told by the oculists and ovariotomists that none should dare attempt an operation for the removal of cataract or ovarian tumor, except the specialist of large experience.

Now, gentlemen, these men of vast experience cannot live always, and who, I would ask, are to be their successors? It reminds me of the advice of the innocent old lady to her son, "That he should never go in the water until he had become an expert swimmer."

